# *MFN2 *point mutations occur in 3.4% of Charcot-Marie-Tooth families. An investigation of 232 Norwegian CMT families

**DOI:** 10.1186/1471-2350-11-48

**Published:** 2010-03-29

**Authors:** Geir J Braathen, Jette C Sand, Ana Lobato, Helle Høyer, Michael B Russell

**Affiliations:** 1 Faculty Division Akershus University Hospital, University of Oslo, Nordbyhagen, Oslo, Norway; 2 Head and Neck Research Group, Research Centre, Akershus University Hospital, Lørenskog, Oslo, Norway; 3 Department of Laboratory Medicine, Section of Medical Genetics, Telemark Hospital, Skien, Norway

## Abstract

**Background:**

Point mutations in the *mitofusin 2 *(*MFN2*) gene has been identified exclusively in Charcot-Marie-Tooth type 2 (CMT2), and in a single family with intermediate CMT. *MFN2 *point mutations are probably the most common cause of CMT2.

**Methods:**

Two-hundred and thirty-two consecutive unselected and unrelated CMT families with available DNA from all regions in Norway were included. We screened for point mutations in the *MFN2 *gene.

**Results:**

We identified four known and three novel point mutations in 8 unrelated CMT families. The novel point mutations were not found in 100 healthy controls. This corresponds to 3.4% (8/232) of CMT families have point mutations in the *MFN2 *gene. The phenotypes were compatible with CMT1 in two families, CMT2 in four families, intermediate CMT in one family and distal Hereditary Motor Neuropathy (dHMN) in one family. This corresponds to 2.3% of CMT1, 5.5% of CMT2, 12.5% of intermediate CMT and 6.7% of dHMN families have a point mutation in the *MFN2 *gene. Point mutations in the *MFN2 *gene is likely to be the fourth most common cause to CMT after duplication of the peripheral myelin protein 22 (PMP22) gene, and point mutations in the Connexin32 (Cx32) and myelin protein zero (MPZ) genes.

**Conclusions:**

The identified known and novel point mutations in the *MFN2 *gene expand the clinical spectrum from CMT2 and intermediate CMT to also include possibly CMT1 and the dHMN phenotypes. Thus, genetic analyses of the *MFN2 *gene should not be restricted to persons with CMT2.

## Background

Charcot-Marie-Tooth (CMT) disease is a heterogeneous group of inherited peripheral neuropathies with an estimated prevalence of 1 in 2,500[1]. At present 40 different genes has been identified to cause inherited peripheral neuropathies[2]. CMT is subdivided into type 1 and 2, depending on whether the motor conduction velocity (MCV) is less or above 38 m/s[3,4]. A third form has intermediate MCV[5]. Intermediate CMT is defined as MCVs between 25 and 45 m/s. Distal Hereditary Motor Neuropathy (dHMN) is the spinal form of CMT[6].

Twelve different genes have been described to cause CMT2. The majority of the mutations are reported in the *mitofusin 2 *(*MFN2*) gene[2]. This gene is responsible for 18-23% of those with CMT2, and is probably the most common cause of CMT2 [7-9]. A *MFN2 *mutation has also been described in a single family with intermediate CMT[9].

Mitofusins are evolutionary conserved GTPases of the mitochondrial outer membrane, and has an essential role for the controlled fusion of the mitochondria membrane[10]. The MFN2 protein spans the mitochondrial outer membrane with a large N-terminal and a relatively short C-terminal exposed to the cytosol[11]. The *MFN2 *mutation so far has almost exclusively been missense mutations that affect the N- or the C-terminal of the MFN2 protein[2,9].

We analyzed 232 Norwegian CMT families for mutations in the *MFN2 *gene.

## Methods

### Patients

Two-hundred and thirty-two consecutive unselected and unrelated CMT families with available DNA from all regions in Norway were included. The total Norwegian population is 4.8 millions people. Based on clinical and neurophysiological data, 86 families had CMT1 (47.3%), 73 families had CMT2 (40.1%), 8 families had intermediate CMT (4.4%) and 15 families had dHMN (8.2%). The neurophysiological phenotype was unknown in 50 families.

### Clinical interview, genetic and neurological examination

Probands and their relatives had a semi-structured clinical interview, a genetic and a neurological examination by geneticist and neurologist GJB. The diagnosis CMT was based on clinical information and exclusion of acquired causes of neuropathy such as endocrine, immunologic, infectious, metabolic, nutritional, toxic drugs and substances, and connective tissue and paraneoplastic disorders. Other hereditary disorders with neuropathy such as Friedreich's ataxia, hereditary spastic paraplegia, myotonic dystrophy etc. were also excluded. All findings were recorded in a neuropathy scheme that has been used and evaluated in Norway [12-14]. This scheme had some supplementary items added by GJB. Cranial nerves, muscle weakness, reflexes and sensation was scored according to the Neuropathy Impairment Score (NIS)[15]. Muscle weakness includes neck flexion, respiratory muscles, and 9 muscle groups in each upper extremity (UE) and 8 muscle groups in each lower extremity (LE). The muscle strength is scored linearly, i.e. normal strength is scored 0 and paralysis is scored 4, while 25%, 50% and 75% muscle weakness are scored 1, 2 and 3.

### Neurophysiology

Standard techniques with surface electrodes were used for antidromic sensory conduction and orthodromic motor conduction. Needle electrodes were used, if the surface electrodes failed to record conduction velocities. Electromyography (EMG) was performed.

### Mutation analysis

Genomic DNA was extracted from white blood cells using FlexiGene DNA Kit (QIAGEN). The coding exons were derived from the published sequences in National Center for Biotechnology Information (NP_055689 and NM_014874, *MFN2*). Primers for PCR amplification of exons and intronic splice junctions were designed with Primer3[16]. Fifty ng of patient genomic DNA was used for the analysis. Each region was amplified in 35 cycles. The sequencing was carried out using the PCR primers, internal sequencing primers when needed, and BigDye Terminator kit version 1.1. Sequencing was performed using Applied Biosystems ABI3130x1 Genetic analyzer and aligned with the Sequencher programme (Gene Codes Corporation, Ann Arbor, MI). The numbering of nucleotides is according to the open reading frame of the cDNA sequences[17]. In families in which a *de novo *point mutation was suspected, paternity was confirmed using 17 STR markers (CSF1PO, D3S1358, D5S818, D7S820, D8S1179, D12S392 D13S317, D16S539, D17S906, D18S51, D21S11, FGA, Penta D, Penta E, TH01, TPOX, and vWA) and Amelogenin. One-hundred persons without neuropathy were screened as controls for sequence variations. For further details see Additional file 1.

We analyzed for mutations consecutively in the genes; *peripheral myelin protein 22 (PMP22), Connexin32 (Cx32), myelin protein zero (MPZ), small integral membrane protein of lysosome/late endosome (SIMPLE), early growth response 2 (EGR2), and MFN2*. The PMP22 duplication was investigated by Real-time quantitative PCR[18]. Coding exons were derived from published sequences in GenBank (*Cx32 (GJB1*): NM_000166, *EGR2*: NM_000399, *MFN2*: BC017061, *MPZ*: NM_000530, *PMP22*: NM_000304 and *SIMPLE*_NM_004862, AB034747. Primers for PCR amplification of exons and intronic splice junctions were designed with Primer3[16].

### Ethics

The study was approved by the ethical committees. Participation was based on informed consent.

## Results

### Molecular genetic analysis

**Figure 1:**
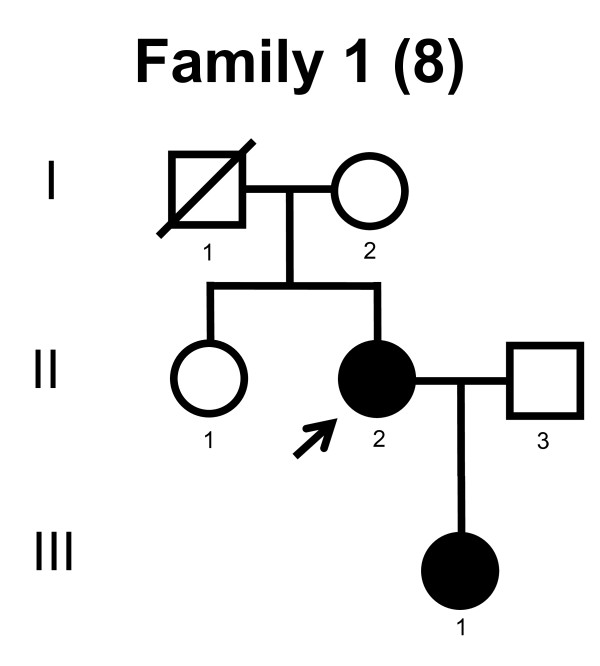
**Pedigree of family 1 with point mutation in the *MFN2 *gene**.

**Figure 2:**
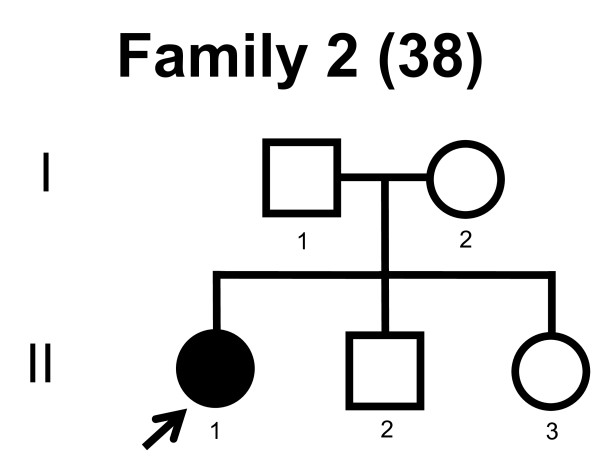
**Pedigree of family 2 with point mutation in the *MFN2 *gene**.

**Figure 3:**
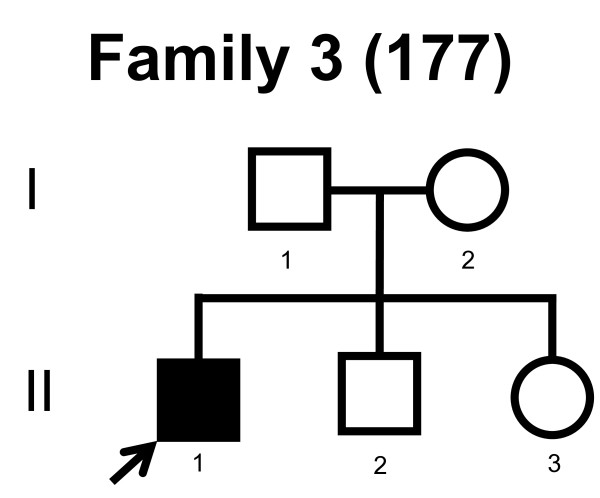
**Pedigree of family 3 with point mutation in the *MFN2 *gene**.

**Figure 4:**
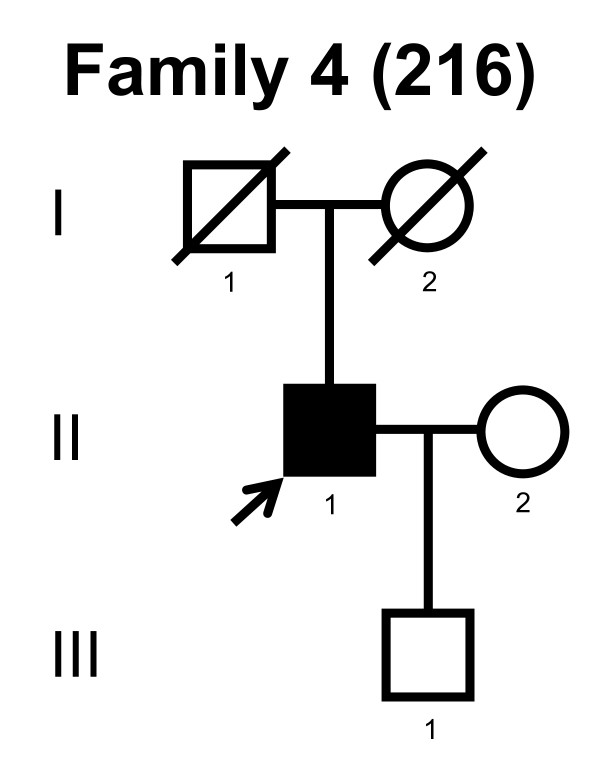
**Pedigree of family 4 with point mutation in the *MFN2 *gene**.

**Figure 5:**
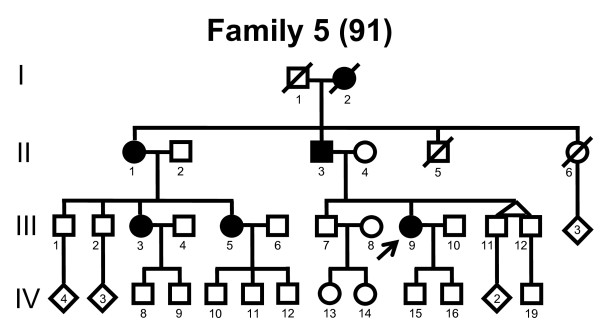
**Pedigree of family 5 with point mutation in the *MFN2 *gene**.

**Figure 6:**
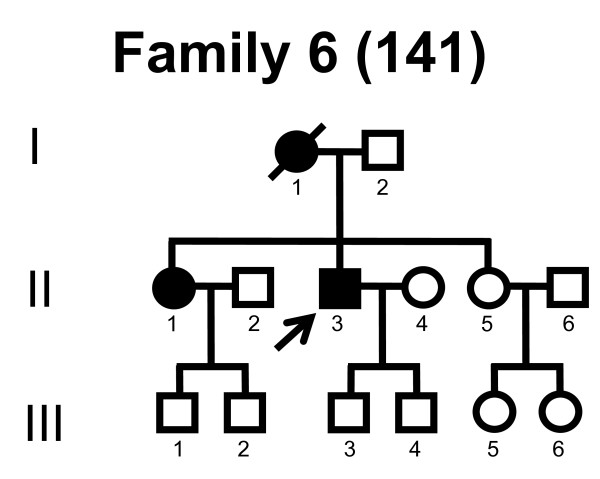
**Pedigree of family 6 with point mutation in the *MFN2 *gene**.

**Figure 7:**
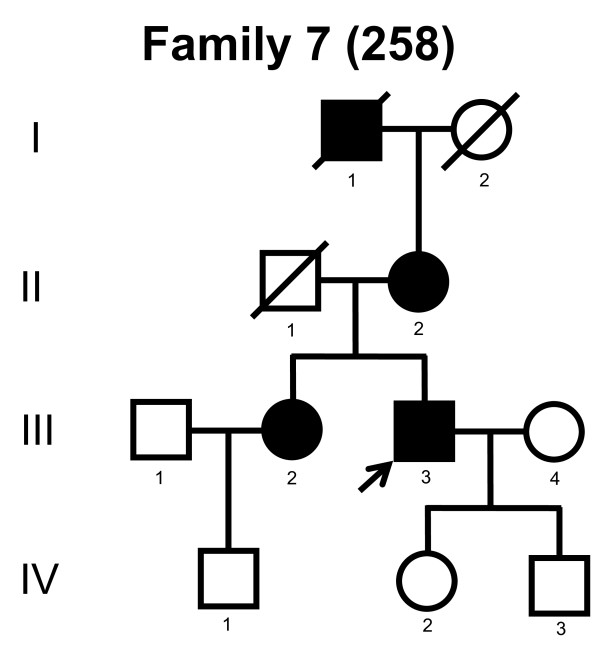
**Pedigree of family 7 with point mutation in the *MFN2 *gene**.

**Figure 8:**
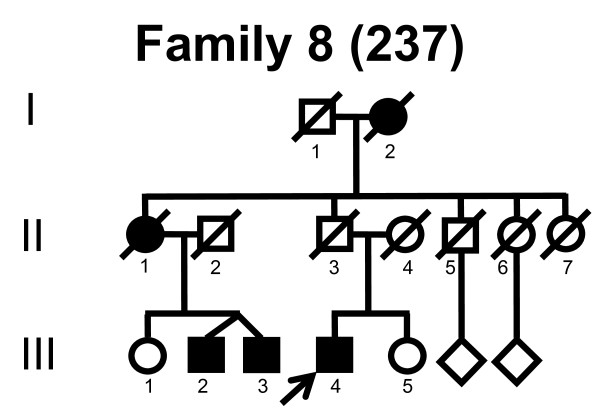
**Pedigree of family 8 with point mutation in the *MFN2 *gene**.

We screened the entire coding region and the intron/exon boundaries of the *MFN2 *gene. A total of 232 unrelated Norwegian families with CMT were included. We identified 7 point mutations in 8 CMT families (Table 1). Four point mutations were known, and three point mutations were novel. All our point mutations were missense mutation that caused an amino acid change. The point mutations were located in exons 4, 14, 15 and 18. The novel point mutations c.1709A>G and c.2119C>T were found in the probands, while c.2146G>A was found in the proband and his affected first cousin. The pedigrees, family 1, 2, 3, 4, 5, 6, 7 and 8 are shown in figure 1, 2, 3, 4, 5, 6, 7 and 8, respectively. Five pedigrees had a segregation pattern compatible with autosomal dominant inheritance (Figures 1, 5, 6, 7 and 8). In four probands the *MFN2 *point mutation occurred *de novo*. Paternity was confirmed in one pedigree (Figure 2). HLA haplotyping was compatible with paternity in another family (Figure 1), while DNA was unavailable in two fathers (Figure 3 and 4). None of the novel point mutations was found in 200 control chromosomes of healthy Norwegians. However, we found a c.1569C>T polymorphism in eight persons, that did not change the amino acid sequence (Ser523Ser). We found no duplication in *PMP22 *gene, and no point mutations in the *PMP22*, *Cx32*, *MPZ*, *SIMPLE *or *EGR2 *genes in the affected with *MFN2 *missense mutations.

**Table 1:** Gender, age at onset, initial symptoms of Charcot-Marie-Tooth disease, phenotype and point mutations in the eight unrelated Norwegian families with mutation in the *MFN2 *gene.

**Family no**.	Family member	Gender	Age at onset (yrs)	Initial symptoms	Phenotype	Point mutation	Exon	Amino acid Change
1	II-2	♀	4	Weakness in legs	CMT1	c.280C>T	4	Arg94Trp
	*III-1*	♀	*4*	*Weakness in legs and pes cavus*				
2	II-1	♀	4	Stumble and dorsal motion weakness in toes	CMT2	c.281G>A	4	Arg94Gln
3	II-1	♂	2	Foot deformity and stumbled	CMT1	c.1403G>A	14	Arg468His
4	II-1	♂	63	Weakness in the right foot, and difficulties walking	dHMN	*c.1709A>G*	15	Asn570Ser
5	III-9	♀	10	Weakness in legs and pes cavus	CMT2	c.2113G>A	18	Val705Ile
6	II-3	♂	47	Paresthesia in feet	CMT2	c.2113G>A	18	Val705Ile
7	III-3	♂	44	Muscular pain during exercise in left leg	CMT2	*c.2119C>T*	18	Arg707Trp
8	*III-2*	♂	*23*	*Recurrent ankle sprains, weakness and reduced balance*	Intermediate CMT	*c.2146G>A*	18	Ala716Thr
	III-4	♂	50	Paresthesia in foot				

Relatives of probands and novel point mutations are shown in *italics*.

### Clinical and neurophysiological findings

Table 2 shows a summary of the neurophysiology and molecular genetic findings in 232 unrelated Norwegian CMT families. We identified a *MFN2 *point mutation in 3.4% (8/232) of the CMT families. The phenotype was compatible with CMT1 in two probands, CMT2 in four probands, intermediate CMT in one proband, and distal Hereditary Motor Neuropathy (dHMN) in one proband. This corresponds to 2.3% of CMT1, 5.5% of CMT2, 12.5% of intermediate CMT and 6.7% of dHMN families have a point mutation in the *MFN2 *gene.

**Table 2:** Summary of neurophysiology and *MFN2 *mutations in 232 CMT families.

Phenotype	Neurophysiology % (n)	*MFN2 *mutations % (frequency)
CMT1	37.1 (86)	2.3 (2/86))
CMT2	31.5 (73)	5.5 (4/73)
Intermediate CMT	3.4 (8)	12.5 (1/8)
dHMN	6.5 (15)	6.7 (1/15)
CMT neurophysiology unknown	21.6 (50)	0 (0/50)

Total	100 (232)	3.4 (8/232)

The mean age at onset was 3.0 years in the CMT1 probands and 26.3 years in the CMT2 probands, while the probands with intermediate CMT and dHMN had late onset (Table 1). The initial symptoms were muscle weakness, difficulties walking and frequent falls. Pes cavus, hammertoes and muscle wasting in feet and legs was frequently observed (Additional file 2, table S3). The sensory modality touch and pain were mildly affected. Vibration and proprioception in the toes, Achilles and patellar reflexes and Romberg was frequently affected. Muscle weakness in the lower limbs varied considerable (Additional file 3, table S4). One proband was wheelchair bound, while her daughter had severe difficulties walking (Family 1). Additional features such as tremor in arm/leg and kyphoscoliosis were sometimes present (Additional file 2, table S3).

The proband in family 4 had a complex history prior to weakness of the right foot and walking difficulties at age 63 years. At age 57 years he had a L4 compression fracture causing spinal stenosis and intermittent claudicatio, until he had a laminectomy, facetectomy and discectomy two years later. A 1/2 year later he experienced lumbar pain and paresthesia in the thighs and had a new laminectomy at age 63 years. One year prior he had bilateral arthroplasty due to osteoarthrosis. He was operated for an abdominal aortic aneurism at age 65 years and four years later he had radiotherapy for a prostate cancer.

Neurophysiological data were available from eight probands and one relative (Additional file 3, table S4). The conduction velocities (CV) were reduced in the probands with the CMT1 phenotypes (Family 1 and 3). The slight reduction in peroneal motor nerve CV within four years could be compatible with demyelination, but we interpreted it as CMT2, see discussion for details (Family 2). The proband in family 4 had normal motor and sensory CV and chronic denervation compatible with dHMN. The CV were slightly reduced but >38 m/s in the probands with CMT2 phenotypes (Family 5-7). The range of the CV in family 8 is compatible with intermediate CMT phenotype. The compound motor action potential (CMAP) and sensory nerve action potential (SNAP) was generally reduced or could not be elicited.

## Discussion

We screened 232 unselected and unrelated Norwegian CMT families with available DNA. A total of 7 *MFN2 *missense mutations were identified in 8 families (Table 1). The literature describes three nonsense mutations and one deletion, but missense mutations in far the most common types of mutations in the *MFN2 *gene[2,9,19].

We identified 4 *de novo *mutations in the *MFN2 *gene, paternity could not be confirmed in two families (family 3 and 4) due to lack of paternal DNA. Our 25-50% (2-4/8) frequency of *de novo *mutations in an population sample corresponds well with the 34% and possibly 55% frequency found in a major collaboration on *MFN2 *mutations[2]. Two of our *de novo *missense mutation occurred in codon 94. The literature describes 5 other families with *de novo *mutations in codon 94 with confirmed paternity[9,20]. These mutations have also been described in families with different ethnicity[2]. This strongly suggest that codon 94 is a hot spot for mutations. The *de novo *c.1403G>A mutation has previously been described in three CMT families, but it was also found in a healthy control[2,21,22]. The c.2113G>A was found in two unrelated Norwegian families, and has also been described by others[2,21]. The c.1709A>G in exon 15 was a novel missense mutations and a *de novo *mutation found in the proband. Mutations in exon 15 have not previously been described. The other novel missense mutations were found in probands from CMT families with several affected, but we only had DNA from a single first cousin whom also carried the mutation. We excluded duplication of PMP22 and point mutation in *PMP22*, *Cx32*, *MPZ*, *SIMPLE *and *EGR2 *genes in the affected with *MFN2 *missense mutations.

Previous mutations in the *MFN2 *gene have all been assigned to a CMT2 phenotype, except from a single family that was assigned intermediate CMT[2,7-9,19-21]. Additional symptoms such as ataxia, optic neuritis, optic atrophy, pyramidal signs, scoliosis and tremor have also been described[2,23-25].

Missense mutations in codon 94, i.e. amino acid change Arg94Trp and Arg94Gln cause intermediate CMT, CMT2 or CMT2 with additional features[9]. The proband in the family with intermediate CMT had motor CV <38 m/s in all tested motor nerves including median nerve and should therefore be classified CMT1. However, his affected father had motor CV >38 m/s, and the family was classified intermediate CMT. The missense mutation Arg94Trp occurred de novo in our family 1. Since we had no neurophysiological data on the probands affected daughter, we classified the family CMT1 rather than the more infrequent intermediate CMT. The missense mutation in codon 468, i.e amino acid change Arg468His was found in a proband with CMT2 and her father with Parkinson disease and distal neuropathy indicating co-segregation as well as in 1 of 260 control chromosomes[21]. A patient with a severe CMT carried two mutations, the Arg468His in *MFN2 *and Gln95stop in *ganglioside-induced differentiation-associated protein 1 *(*GDAP1*)[22]. Two family members carried exclusively the missense mutation in the *MFN2 *that caused the amino acid change Arg468His. One had a mild axonal neuropathy at age 56 years, while the other was unaffected at age 18 years[22]. Three other family members with the Gln95stop in *GDAP1 *were unaffected. The proband in our family 3 with the Arg468His amino acid change had reduced motor CV, while 1 of 250 control chromosomes had the missense mutation carried by an unaffected woman age 25 years.

We analyzed all types of CMT families and found *MFN2 *missense mutation in CMT1, CMT2, intermediate CMT and dHMN patients.

The CV in the median motor nerve is used as reference for classifying CMT1, CMT2 and intermediate CMT [3-5]. dHMN is defined with normal motor and sensory CV and chronic denervation on EMG[6]. We do not have CV of the median motor nerve in the probands with CMT1. However, they all had reduced CV in motor nerves compatible with CMT1. The CV is equal in the ulnar and the median motor nerve. Thus, the proband with CV <38 m/s in the ulnar motor nerve was classified CMT1 (Family 1). We interpreted the marked reduction in motor CV of the tibial nerve as demyelination (Family 3). The slight reduction in motor CV of the peroneal nerve in Family 2 was interpreted as CMT2, as the CV cut off for the normal value is less in the peroneal than the median nerve, and an extrapolation with this proportional factor would increase the CV from 35.2 to 42.1 m/s, while a similar extrapolation in family 3 would increase the CV from 15.7 to 18.8 m/s (Additional file 2, table S3). Our neurophysiological data is far from optimal. The 232 consecutive unselected and unrelated CMT families with available DNA were from all regions in Norway, and were included into the study irrespective of the perfection of neurophysiological parameters, if the clinical or other parameters suggested it was CMT. An International study of mutations in the *MFN2*, included 323 unrelated probands of whom 44 unrelated probands had an unknown neurophysiology[9]. The lack of complete neurophysiological data makes the neurophysiological classification a challenge, along with the fact that CMAP were reduced in several recordings. Severely reduced CMAP and low CV in lower limbs suggest CMT2. However, the neurophysiological classification on CMT is based on CV alone not including CMAP or other neurophysiological features. For that reason we focused on CV in our interpretation. This provides a challenge in family 2, 3, 4 and 5 since neurophysiological data is only available from the lower limbs. Family 4 is diagnosed as dHMN based on a motor and a sensory CVs were normal, but the amplitudes of the sensory potentials were not measured which makes it difficult to judge whether the sensory nerves were involved or not.

The International study on *MFN2 *mentioned above included 323 unrelated probands, of whom 249 (77%) probands were diagnosed as CMT2, 20 (6%) had intermediate CMT, six (2%) had CMT1, three (1%) had dHMN and one (<1%) had hereditary sensory and autonomic neuropathy (HSAN, while 44 (14%) had an unknown neurophysiological phenotype. *MFN2 *mutations were found in 28 probands with CMT2 and in one family with intermediate CMT. We found the *MFN2 *mutation in 2 of 86 (2.3%) unrelated CMT1 families and in 1 of 15 (6.7%) unrelated dHMN families. These low frequencies of *MFN2 *mutations in CMT1 and dHMN made it unlikely for the International study to obtain *MFN2 *mutations in these CMT subtypes. Unfortunately, we do not have CV from the median nerve in the two families classified as CMT1. Thus, further studies are needed to confirm that *MFN2 *mutations can cause CMT1.

Clinically the age at onset was early in the CMT1 families (Family 1 and 3), while it was late in CMT2, intermediate and dHMN (Family 4-8), with one exception (Family 2). Additional symptoms such as ataxia, kyphoscoliosis and tremor were found three families (Table 2). The phenotype genotype correlation can be questioned in the proband with dHMN, since the classification was based on single motor and sensory nerve and he had multiple other disorders that might have caused his motor and sensory symptoms (for details see result section). However, since the motor and sensory CVs were normal, we characterized the proband as dHMN. The identified missense mutation c.1709A>G in exon 15 might be a polymorphism, although we did not find the mutation among 200 control chromosomes.

Point mutations in the *MFN2 *gene is likely to be the fourth most common cause to CMT after the duplication of *PMP22 *gene, and point mutations in the *Cx32 *and *MPZ *genes. We found that 2.3% of CMT1, 5.5% of CMT2, 12.5% of intermediate CMT and 6.7% of dHMN families have a point mutation in the *MFN2 *gene. We analyzed for mutations in five other common genes that may cause CMT1, CMT2, intermediate CMT or dHMN. However the exclusion analyses were not exhaustive since mutations in at least 40 genes can cause CMT. We suggest that genetic analyses of *MFN2 *should not be restricted exclusively to those with a CMT2 phenotype.

## Conclusions

Today only people with CMT2 receive testing for the *MFN2 *gene, as the literature provides no evidence for testing other subtypes of CMT. Our main finding indicates that MFN2 mutations might be found in all subtypes of CMT. Thus, *MFN2 *gene testing should not be restricted to CMT2.

## Competing interests

The authors declare that they have no competing interests.

## Authors' contributions

GJB acquired the material, conceived the study, participated in the design of the study and drafted the manuscript. JCS, AL and HH carried out the molecular genetic studies and the sequence alignment. MBR conceived the study, participated in the design of the study and drafted the manuscript. All authors read and approved the final manuscript.

## Authors' informations

GJB is a neurologist and geneticist. JCS, AL and HH are cand. scient. MBR is professor of neurology, PhD and DMSci. The authors main interest is genetics in neurological disorders with special emphasis on CMT.

## Pre-publication history

The pre-publication history for this paper can be accessed here:

http://www.biomedcentral.com/1471-2350/11/48/prepub

## Supplementary Material

Additional file 1 **Appendix 1**. Primer sequences used for amplifying and sequencing the coding regions of the *MFN2 *gene. Click here for file

Additional file 2 **Table S3**. Clinical characteristics of the patients with point mutations in the *MFN2 *gene. Click here for file

Additional file 3 **Table S4**. Neurophysiology in patients with Charcot-Marie-Tooth disease caused by point mutations in the *MFN2 *gene. Click here for file

## References

[B1] Skre H Genetic and clinical aspects of Charcot-Marie-Tooth's disease Clin Genet 1974 6 98 118 4430158 10.1111/j.1399-0004.1974.tb00638.x

[B2] The Mutation Database of Inherited Peripheral Neuropathies http://www.molgen.ua.ac.be/CMTMutations/default.cfm

[B3] Dyck PJ Lambert EH Lower motor and primary sensory neuron diseases with peroneal muscular atrophy. I. Neurologic, genetic and electrophysiologic findings in hereditary polyneuropathies Arch Neurol 1968 18 603 618 4297451 10.1001/archneur.1968.00470360025002

[B4] Harding AE Thomas PK The clinical features of hereditary motor and sensory neuropathy types I and II Brain 1980 103 259 280 10.1093/brain/103.2.259 7397478

[B5] Nicholson G Nash J Intermediate nerve conduction velocities define X-linked Charcot-Marie-Tooth neuropathy families Neurology 1993 43 2558 2564 8255457 10.1212/wnl.43.12.2558

[B6] Harding AE Dyck PJ, Thomas PK Inherited Neuronal Atrophy and degeneration predominantly of lower motor neurons Peripheral Neuropathy 2005 4 Philadelphia: Elsevier, Saunders 1603 1621

[B7] Kijima K Numakura C Izumino H Mitochondrial GTPase mitofusin 2 mutation in Charcot-Marie-Tooth neuropathy type 2A Hum Genet 2005 116 23 27 10.1007/s00439-004-1199-2 15549395

[B8] Lawson VH Graham BV Flanigan KM Clinical and electrophysiologic features of CMT2A with mutations in the mitofusin 2 gene Neurology 2005 65 197 204 10.1212/01.wnl.0000168898.76071.70 16043786

[B9] Verhoeven K Claeys KG Züchner S MFN2 mutation distribution and genotype/phenotype correlation in Charcot-Marie-Tooth type 2 Brain 2006 129 2093 2102 10.1093/brain/awl126 16714318

[B10] Santel A Get the balance right: mitofusins roles in health and disease Biochim Biophys Acta 2006 1763 490 499 10.1016/j.bbamcr.2006.02.004 16574259

[B11] Rojo M Legros F Chateau D Membrane topology and mitochondrial targeting of mitofusins, ubiquitous mammalian homologs of the transmembrane GTPase Fzo J Cell Sci 2002 115 1663 1674 11950885 10.1242/jcs.115.8.1663

[B12] Skre H Hereditary ataxias and their associated traits. A clinical, genetic, and epidemiological study in Western Norway 1976 Bergen and Oslo: University of Bergen and University of Oslo (Thesis) 1 288

[B13] Skre H Neurological signs in a normal population Acta Neurol Scand 1972 48 575 606 10.1111/j.1600-0404.1972.tb07577.x 4643256

[B14] Skre H Application of a quantitative scoring system in the investigation of some hereditary neurological disorders Clin Genet 1974 5 163 172 4829426 10.1111/j.1399-0004.1974.tb01678.x

[B15] Dyck PJ Turner DW Davies JL Electronic case-report forms of symptoms and impairments of peripheral neuropathy Can J Neurol Sci 2002 29 258 266 12195616 10.1017/s0317167100002043

[B16] Rozen S Skaletsky HJ Krawetz S, Misener S Primer3 on the WWW for general users and for biologist programmers Bioinformatics Methods and Protocols: Methods in Molecular Biology 2000 Totowa, NJ: Humana Press 365 386 http://fokker.wi.mit.edu/primer3 10.1385/1-59259-192-2:365 10547847

[B17] den Dunnen JT Antonarakis SE Nomenclature for the description of human sequence variations Hum Genet 2001 109 121 124 10.1007/s004390100505 11479744

[B18] Aarskog NK Vedeler CA Real-time quantitative polymerase chain reaction. A new method that detects both the peripheral myelin protein 22 duplication in Charcot-Marie-Tooth type 1A disease and the peripheral myelin protein 22 deletion in hereditary neuropathy with liability to pressure palsies Hum Genet 2000 107 494 498 10.1007/s004390000399 11140948

[B19] Züchner S Mersiyanova IV Muglia M Mutations in the mitochondrial GTPase mitofusin 2 cause Charcot-Marie-Tooth neuropathy type 2A Nat Genet 2004 36 449 451 10.1038/ng1341 15064763

[B20] Neusch C Senderek J Eggermann T Mitofusin 2 gene mutation (R94Q) causing severe early onset axonal polyneuropathy (CMT2A) Eur J Neurol 2007 14 575 577 10.1111/j.1468-1331.2006.01688.x 17437620

[B21] Engelfried K Vorgerd M Hagedorm M Charcot-Marie-Tooth neuropathy type 2A:novel mutations in the mitofusin 2 gene (MFN2) BMC Med Genet 2006 7 53 10.1186/1471-2350-7-53 16762064 PMC1524942

[B22] Banchs I Casasnovas C De Jorge L A novel mutation in GDAP1 and a change in MFN2 gene in a family with a severe form of Charcot-Marie-Tooth Eur J Hum Genet 2008 16 suppl 2 S88

[B23] Zhu D Kennerson MI Walizada G Charcot-Marie-Tooth with pyramidal signs is genetically heterogeneous:families with and without MFN2 mutations Neurology 2005 65 496 497 10.1212/01.wnl.0000171345.62270.29 16087932

[B24] Chung KW Kim SB Park KD Early onset severe and late-onset mild Charcot-Marie-Tooth disease with mitofusin 2 (MFN2) mutations Brain 2006 129 2103 2118 10.1093/brain/awl174 16835246

[B25] Züchner S De Jonghe P Jordanova A Axonal neuropathy with optic atrophy (HMSN VI) is caused by mutations in mitofusin 2 Ann Neurol 2006 59 276 281 10.1002/ana.20797 16437557

