# Supplementary material for: MFN2 point mutations occur in 3.4% of Charcot-Marie-Tooth families. An investigation of 232 Norwegian CMT families
Source: BMC Med Genet. 2010 Mar 29;11:48. doi: 10.1186/1471-2350-11-48 (PMC2859816; doi:10.1186/1471-2350-11-48)
Supplement: Additional file 1 — Appendix 1. Primer sequences used for amplifying and sequencing the coding regions of the MFN2 gene. [file 1471-2350-11-48-S1.DOC]

Appendix 1. Primer sequences used for amplifying and sequencing the coding regions of the *MFN2* gene.

| **Exon number** | **Primer**  **Direction** | **Primer sequence** | **Enzyme^1^** | **Annealing temperature (ºC)** |
| --- | --- | --- | --- | --- |
| 3 | Forward | ggttcgctcacgttagcttc | Yes | 57**º** |
| 3 | Reverse | cgcaaatcccactagctaaa | Yes | 57**º** |
| 4 | Forward | tggtgacccattttcaatcc | Yes | 57**º** |
| 4 | Reverse | tggaacgttctgtgaccttg | Yes | 57**º** |
| 5 | Forward | tactggtggctttgctgaca | Yes | 57**º** |
| 5 | Reverse | agcaggcacagggctgac | Yes | 57**º** |
| 6 | Forward | ggttcctcctcagcctcttt | Yes | 57**º** |
| 6 | Reverse | agggaccctggcctagatta | Yes | 57**º** |
| 7 | Forward | gtcccaggtctgttctcagc | Yes | 57**º** |
| 8 | Reverse | cactagatccaggggtgcag | Yes | 57**º** |
| 9 | Forward | gggccacctacactcactct | Yes | 57**º** |
| 10 | Forward^2^ | cttggtttctggggatttca |  |  |
| 10 | Reverse^2^ | cacagaatcgccagatacca |  |  |
| 11 | Reverse | cttgtctcggcagctctctc | Yes | 57**º** |
| 12 | Forward | tggatttctcaccagtactctgc | Yes | 57**º** |
| 12 | Reverse^2^ | ggaggtctgctttcttctgg |  |  |
| 13 | Forward^2^ | aacagtgtgcttccttttgc |  |  |
| 14 | Reverse | ccacagctgcccagttcc | Yes | 57**º** |
| 15 | Forward | atccctggcagtagctggta | Yes | 57**º** |
| 15 | Reverse | tctggaggcagggtacagac | Yes | 57**º** |
| 16 | Forward | cccagactagggcaacactg | Yes | 59**º** |
| 16 | Reverse^2^ | ccatgcaatcacagaggaga |  |  |
| 17 | Forward^2^ | gaaacatgaaggctccttgg |  |  |
| 17 | Reverse | cctaaaggaagtcccctgct | Yes | 59**º** |
| 18 | Forward | ctgggtcccttctctctcct | Yes | 57**º** |
| 18 | Reverse | accctggagccctaacctt | Yes | 57**º** |
| 19 | Forward | cctggcgggtagtcctaata | Yes | 57**º** |
| 19 | Reverse | gagcccacatggcacttag | Yes | 57**º** |
|  |  |  |  |  |

^1^Eppendorf hotmaster taq polymerase.

^2^Primer only used for sequencing.
