# Supplementary material for: MFN2 point mutations occur in 3.4% of Charcot-Marie-Tooth families. An investigation of 232 Norwegian CMT families
Source: BMC Med Genet. 2010 Mar 29;11:48. doi: 10.1186/1471-2350-11-48 (PMC2859816; doi:10.1186/1471-2350-11-48)
Supplement: Additional file 2 — Table S3. Clinical characteristics of the patients with point mutations in the MFN2 gene. [file 1471-2350-11-48-S2.DOC]

Table S3. Clinical characteristics of the patients with point mutations in the *MFN2* gene.

| **Family number** | **1** | | **2** | **3** | **4** | **5** | **6** | **7** | **8** | |
| --- | --- | --- | --- | --- | --- | --- | --- | --- | --- | --- |
| Family member | II-2 | III-1 | II-1 | II-1 | II-1 | III-9 | II-3 | III-3 | III-2 | III-4 |
| Gender | ♀ | ♀ | ♀ | ♂ | ♂ | ♀ | ♂ | ♂ | ♂ | ♂ |
| Age at onset | 4 | 4 | 4 | 2 | 63 | 10 | 47 | 44 | 23 | 50 |
| Disease duration | 41 | 13 | 5 | 14 | 7 | 32 | 14 | 8 | 48 | 15 |
| *Clinical characteristics* | |  |  |  |  |  |  |  |  |  |
| Muscle wasting^1^ | |  |  |  |  |  |  |  |  |  |
| Underarm | 2 | 1 | 0 | 1 | 0 | 0 | 0 | 0 | 1 | 0 |
| Hand | 2 | 2 | 0 | 1 | 0 | 0 | 0 | 0 | 2 | 0 |
| Thigh | 2 | 2 | 0 | 0 | 0 | 1/0^2^ | 0 | 0 | 1 | 0/1^2^ |
| Leg | 2 | 2 | 1 | 0 | 2/1^2^ | 2/1^2^ | 0 | 2 | 1 | 0/1^2^ |
| Feet | 2 | 2 | 1 | 1 | 2 | 2 | 0 | 2 | 2 | 1 |
| Muscle weakness^NIS^ | |  |  |  |  |  |  |  |  |  |
| Elbow extension | 0 | 0 | 0 | 0 | 1/0^2^ | 0 | 0 | 0 | 1 | 0 |
| Wrist flexion | 3,25 | 0 | 0 | 0 | 0 | 0 | 0 | 0 | 0 | 0 |
| Wrist extension | 3,25 | 0 | 0 | 0 | 0 | 0 | 0 | 0 | 0 | 0 |
| Finger flexion | 3,25 | 2 | 0 | 0 | 0 | 0 | 0 | 0 | 2 | 1 |
| Finger spread | 3,25 | 2 | 0 | 1 | 0 | 0 | 0 | 0 | 2 | 1 |
| Thumb abduction | 3,25 | 2 | 0 | 0 | 1/0^2^ | 0 | 0 | 1/0^2^ | 2 | 1 |
| Knee flexion | 3,25 | 3 | 0 | 0 | 0 | 0 | 0 | 0 | 0 | 0 |
| Knee extension | 3,5 | 3 | 0 | 0 | 0 | 0 | 0 | 0 | 1 | 0 |
| Ankle dorsiflexors | 4 | 3,5 | 3,75 | 1 | 3,25 | 1 | 0 | 2 | 3,75 | 0 |
| Ankle plantar flexors | 4 | 3,5 | 0 | 0 | 3,25 | 0 | 0 | 2 | 3,75 | 0 |
| Toe extensors | 4 | 4 | 3,75 | 2 | 3 | 1 | 1 | 2 | 3,75 | 1 |
| Toe flexors | 4 | 4 | 0 | 1 | 3 | 0 | 0 | 2 | 3,75 | 1 |
| Sensory loss | |  |  |  |  |  |  |  |  |  |
| *Touch*^1^ | |  |  |  |  |  |  |  |  |  |
| Feet, leg | 1 | 0 | 0 | 0 | 0 | 0 | 0 | 1 | 1 | 0 |
| Arm, hand | 1 | 0 | 0 | 0 | 0 | 0 | 0 | 0 | 0 | 0 |
| *Pain*^1^ |  |  |  |  |  |  |  |  |  |  |
| Overarm | 0 | 0 | 0 | 0 | 0 | 0 | 0 | 0 | 1 | 0 |
| Hand, underarm | 1 | 0 | 0 | 0 | 0 | 0 | 0 | 0 | 1 | 1 |
| Thigh | 0 | 0 | 0 | 0 | 0 | 0 | 0 | 0 | 1 | 1 |
| Feet, leg | 1 | 0 | 0 | 1 | 1 | 0 | 0 | 1 | 2 | 2 |
| *Vibration*^1^ | |  |  |  |  |  |  |  |  |  |
| Hand | 1 | 0 | 0 | 0 | 0 | 1 | 0 | 0 | 0 | 1 |
| Knee | 1 | 1 | 0 | 1 | 0/1^2^ | 1 | 0 | 0 | 1 | 1 |
| Ankle | 2 | 1 | 0 | 1 | 2 | 1 | 0 | 2 | 2 | 2 |
| 1. metatarsal | 2 | 1 | 0 | 1 | 2 | 2 | 0 | 2 | 2 | 2 |
| 1.toe | 2 | 1 | 0 | 1 | 2 | 2 | 0 | 2 | 2 | 2 |
| *Proprioceptive*^1^ | |  |  |  |  |  |  |  |  |  |
| Toe | 2 | 0 | 0 | 1 | 2 | 0 | 0 | 0 | 2 | 2 |
| Reflexes^1^ | |  |  |  |  |  |  |  |  |  |
| Biceps | 1 | 0 | 0 | 2 | 0 | 0 | 2 | 0 | 2 | 2 |
| Triceps | 1 | 0 | 0 | 0 | 0 | 0 | 0 | 0 | 2 | 0/1^2^ |
| Brachioradialis | 2 | 0 | 0 | 0 | 0 | 0 | 0 | 0 | 1 | 1/0^2^ |
| Patellar | 2 | 2 | 0 | 1 | 0 | 0 | 2 | 2 | 2 | 2 |
| Achilles | 2 | 2 | 1 | 2 | 2 | 0 | 2 | 2 | 2 | 2 |
| Deformities^1^ | |  |  |  |  |  |  |  |  |  |
| Pes cavus | 2 | 2 | 2 | 2 | 0 | 2 | 0 | 0 | 0 | 0 |
| Hammertoes | 2 | 0 | 1 | 1 | 0 | 1 | 0 | 1 | 2 | 2 |
| Pes planus | 0 | 0 | 0 | 0 | 0 | 0 | 0 | 0 | 0 | 0 |
| Kyphoscoliosis | 0 | 0 | 0 | 2 | 0 | 0 | 0 | 0 | 0 | 0 |
| Ataxia^1^ |  |  |  |  |  |  |  |  |  |  |
| Arms | 0 | 0 | 0 | 0 | 1 | 0 | 0 | 0 | 1 | 0 |
| Legs | 0 | 0 | 0 | 0 | 1 | 0 | 0 | 0 | 1 | 1 |
| Tremor |  |  |  |  |  |  |  |  |  |  |
| Arms | 0 | 0 | 0 | 0 | 0 | 0 | 0 | 0 | 1 | 0 |
| Legs | 0 | 0 | 0 | 0 | 0 | 0 | 0 | 0 | 1 | 0 |
| Romberg^1^ | 2 | 1 | 0 | 1 | 2 | 0 | 0 | 1 | 2 | 1 |
| **NIS score** | **112** | **64** | **17** | **26** | **37** | **10** | **14** | **29** | **68** | **30** |
|  |  |  |  |  |  |  |  |  |  |  |

^1^0 = normal; 1 = mild/moderate affected modality; 2 = severe affected modality. ^2^Asymmetrical signs right/left. Neuropathy Impairment Score (NIS) for details see material and methods section.
