# Supplementary material for: MFN2 point mutations occur in 3.4% of Charcot-Marie-Tooth families. An investigation of 232 Norwegian CMT families
Source: BMC Med Genet. 2010 Mar 29;11:48. doi: 10.1186/1471-2350-11-48 (PMC2859816; doi:10.1186/1471-2350-11-48)
Supplement: Additional file 3 — Table S4. Neurophysiology in patients with Charcot-Marie-Tooth disease caused by point mutations in the MFN2 gene. [file 1471-2350-11-48-S3.DOC]

Table S4. Neurophysiology in patients with Charcot-Marie-Tooth disease caused by point mutations in the *MFN2* gene.

|  |  |  | |  |  | | | | | | | |  | | | | | |  |
| --- | --- | --- | --- | --- | --- | --- | --- | --- | --- | --- | --- | --- | --- | --- | --- | --- | --- | --- | --- |
|  | Sex | Age at | | R/L | Motor nerves | | | | | | | | Sensory nerves | | | | | | EMG  chronic  dener-  vation |
|  |  | Onset  (yrs) | Exami-nation  (yrs) |  | Median | | Ulnar | | Peroneal | | Tibial | | Median | | Ulnar | | Sural | |  |
|  |  |  |  |  | CMAP | CV | CMAP | CV | CMAP | CV | CMAP | CV | SNAP | CV | SNAP | CV | SNAP | CV |  |
| Normal values → | | |  |  | 4.0 | 49.0 | 4.0 | 49.0 | 3.0 | 41.0 | 3.0 | 41.0 | 12.0 | 46.0 | 17.0 | 47.0 | 17.0 | 44.0 |  |
|  | | |  |  |  |  |  |  |  |  |  |  |  |  |  |  |  |  |  |
| *Family 1* |  |  |  |  |  |  |  |  |  |  |  |  |  |  |  |  |  |  |  |
| II-2 | ♀ | 4 | 38 | L | - | - | **-** | **35.5** | **-** | **-** | **-** | **-** | - | - | - | - | **-** | **23.1** | **Present** |
|  |  |  |  |  |  |  |  |  |  |  |  |  |  |  |  |  |  |  |  |
| *Family 2* |  |  |  |  |  |  |  |  |  |  |  |  |  |  |  |  |  |  |  |
| II-1 | ♀ | 4 | 7 | R | - | - | **-** | **-** | ↓↓ | 43.0 | **-** | **-** | - | - | - | - | **A** | **A** | **Present** |
|  |  |  | 11 | R | - | - | **-** | **-** | ↓↓ | **35.2** | **-** | **-** | **4.4** | - | - | - | **0.6** | **38.1** | - |
|  |  |  |  | L | - | - | **-** | **-** | **-** | **-** | **-** | **-** | - | - | - | - | **0.5** | **39.6** | - |
| *Family 3* |  |  |  |  |  |  |  |  |  |  |  |  |  |  |  |  |  |  |  |
| II-1 | ♂ | 2 | 16 | R | - | - | **-** | **-** | **0.09** | **A** | **0.16** | **15.7** | - | - | - | - | **A** | **A** | **Present** |
|  |  |  |  | L | - | - | **-** | **-** | **A** | **A** | **0.12** | **A** | - | - | - | - | **A** | **A** | - |
| *Family 4* |  |  |  |  |  |  |  |  |  |  |  |  |  |  |  |  |  |  |  |
| II-1 | ♂ | 63 | 61 | L | - | - | **-** | **-** | **-** | 46.9 | **-** | **-** | - | - | - | - | **-** | 54.7 | Normal |
|  |  |  | 66 | R | - | - | **-** | **-** | **-** | 42.0 | **-** | **-** | - | - | - | - | **-** | **-** | **Present** |
|  |  |  |  | L | - | - | **-** | **-** | **-** | 47.6 | - | **-** | - | - | - | - | **-** | 51.9 | **Present** |
| *Family 5* |  |  |  |  |  |  |  |  |  |  |  |  |  |  |  |  |  |  |  |
| III-9 | ♀ | 10 | 49 | R | - | - | - | - | 6.6 | 41.1 | 5.4 | 53.5 | - | - | - | - | **A** | **A** | - |
|  |  |  |  | L | - | - | - | - | 6.4 | 42.6 | 5.6 | 52.4 | - | - | - | - | **1.3** | **42.4** | **Present** |
| *Family 6* |  |  |  |  |  |  |  |  |  |  |  |  |  |  |  |  |  |  |  |
| II-3 | ♂ | 47 | 61 | R | - | **46.9** | **-** | **-** | **A** | **A** | **A** | **A** | - | - | - | - | **A** | **A** | **Present** |
|  |  |  |  | L | - | **46.0** | **-** | **-** | **A** | **A** | **A** | **A** | - | - | - | - | **A** | **A** | - |
| *Family7* |  |  |  |  |  |  |  |  |  |  |  |  |  |  |  |  |  |  |  |
| III-3 | ♂ | 44 | 52 | R | 4.0 | **41.0** | 4.0 | **44.0** | **0.2** | **34.0** | **0.1** | **A** | **5.0** | **44.0** | **3.0** | **44.0** | **A** | **A** | **Present** |
|  |  |  |  | L | - | - | **-** | **-** | **0.1** | **37.0** | **0.05** | **A** | - | - | - | - | **A** | **A** | **-** |
| *Family 8* |  |  |  |  |  |  |  |  |  |  |  |  |  |  |  |  |  |  |  |
| III-2 | ♂ | 23 | 62 | R | 4.0 | 47.0 | **-** | 52.0 | **0.7** | **25.0** | **A** | **A** | **7.0** | 55.0 | **4.0** | **44.0** | **A** | **A** | **Present** |
|  |  |  | 62.1 | R | **3.5** | **44.0** | 9.0 | **39.0** | **A** | **A** | **A** | **A** | **4.0** | 56.0 | **2.0** | **44.0** | **A** | **A** | **Present** |
|  |  |  | 63 | R | 7.8 | **38.0** | 7.4 | **43.0** | **A** | **A** | **A** | **A** | **6.0** | **42.0** | **6.0** | **25.0** | **A** | **A** | **Present** |
| III-4 | ♂ | 50 | 64 | R | **2.0** | **37.0** | **3.2** | **43.0** | **A** | **A** | **0.1** | **21.0** | **A** | **A** | **5.0** | **25.0** | **0.8** | **26.0** | **Present** |
|  |  |  |  | L | - | **-** | - | **-** | **A** | **A** | **0.3** | **31.0** | **-** | **-** | **-** | **-** | **-** | **-** | **-** |
|  |  |  |  |  |  |  |  |  |  |  |  |  |  |  |  |  |  |  |  |

CMAP = compound motor action potential (mV); SNAP = sensory nerve action potential (µV); CV, conduction velocity (m/s); A,absent evoked response; -, not measured; R/L = right/left;

Bold = abnormal values
